# Clinicians approaches to management of background treatment in patients with SLE in clinical remission: results of an international observational survey

**DOI:** 10.1136/lupus-2016-000173

**Published:** 2017-06-29

**Authors:** Pintip Ngamjanyaporn, Eoghan M McCarthy, Jamie C Sergeant, John Reynolds, Sarah Skeoch, Benjamin Parker, Ian N Bruce

**Affiliations:** 1 Arthritis Research UK Centre for Epidemiology, Centre for Musculoskeletal Research, Institute of Inflammation and Repair, The University of Manchester, Manchester Academic Health Science Centre, Manchester, UK; 2 Department of Medicine, Faculty of Medicine Ramathibodi Hospital, Mahidol University, Bangkok, Thailand; 3 NIHR Manchester Musculoskeletal Biomedical Research Unit, Central Manchester University Hospital NHS Foundation Trust, Manchester Academic Health Science Centre, Manchester, UK

**Keywords:** Systemic Lupus Erythematosus, Disease Activity, Corticosteroids, Remission, serology

## Abstract

**Background:**

The definition of remission in systemic lupus erythematosus (SLE) remains unclear, especially how background treatment should be interpreted.

**Objective:**

To determine preferences of clinicians in treatment of patients in clinical remission from SLE and to assess how previous severity, duration of remission and serology influence changes in treatment.

**Methods:**

We undertook an internet-based survey of clinicians managing patients with SLE. Case scenarios were constructed to reflect different remission states, previous organ involvement, serological abnormalities, duration of remission and current treatment (hydroxychloroquine (HCQ), steroids and/or immunosuppressive (ISS) agents).

**Results:**

130 clinicians from 30 countries were surveyed. The median (range) duration of practice and number of patients with SLE seen each month was 13 (2–42) years and 30 (2–200), respectively. Management decisions in all scenarios varied with greater caution in treatment reduction with shorter duration of remission, extent of serological abnormalities and previous disease severity. Even with mild disease, normal serology and a 5-year clinical remission, 113 (86.9%) clinicians continue to prescribe HCQ. Persistent abnormal serology in any scenario led to a reluctance to reduce or discontinue medications. Prescribing in remission, particularly of steroids and HCQ, varied significantly according to geographical location.

**Conclusions:**

Clinicians preferences in withdrawing or reducing treatment in patients with SLE in clinical remission vary considerably. Serological abnormalities, previous disease severity and duration of remission all influence the decision to reduce treatment. It is unusual for clinicians to stop HCQ even after prolonged periods of clinical remission. Any definition(s) of remission needs to take into consideration such evidence on how maintenance treatments are managed.

## Introduction

Systemic lupus erythematosus (SLE) is a prototypical autoimmune disease characterised by alternating periods of disease activity and quiescence. The main aim of treatment is to control inflammatory disease activity and prevent flares of disease while in remission.^1^ The mortality and morbidity associated with SLE have improved significantly over the past 50 years owing to introduction of treatments such as corticosteroids, antimalarial agents (AMs), immunosuppressive (ISS) drugs and more recently, biological agents. Although such treatments have improved disease control, all are associated with potential long-term sequelae that can adversely affect patient outcomes and thus patients should be maintained on the minimum long-term treatment necessary to satisfactorily control disease.^2^ Studies have shown that while up to 17% of patients with SLE may successfully stop all medications for a period of time, only 1% of patients will successfully continue without all medications for ≥5 years and have no clinical or serological disease activity during that time.^3^

Efforts to define particular disease ‘states’ in SLE are of value for physicians looking after patients but also for the research community to enable better stratification of patients, thus allowing researchers to standardise comparisons across cohorts. A few years ago, lupus experts reached a consensus about a definition of lupus flare.^4^ The treat-to-target (T2T) initiative has also provided a framework of principles to consider when treating SLE.^5^ A key recommendation of this initiative is that remission is the key ‘treatment target’ and, if not achievable, then low disease activity should be targeted.^5^ However, at present there is no clear consensus about what constitutes remission in SLE. In particular, there is a lack of agreement about how background treatment should affect any potential definition of remission.

A number of guidelines for the treatment of active SLE exist. In contrast, there is little guidance about how and when to decrease or stop medication in patients with SLE.^5–9^ Physicians' approaches to reducing treatment remain undefined. It is unclear which factors influence this decision (eg, previous disease phenotype, current serology) or the order in which drugs are withdrawn and whether AMs are ever reduced or withdrawn in routine practice. Knowledge of physicians' habits and opinions is important so that any definition of remission can be pragmatically applied both in the clinic and in future studies.

We aimed to survey clinicians caring for patients with SLE to determine how background treatment is managed during clinical remission. In particular, we aimed to study how previous SLE severity, duration of remission and serology influence changes in treatment and to determine the usual order of drug reduction and/or withdrawal in usual care.

## Methods

### Survey creation

The survey was developed by two consultant rheumatologists (INB, BP) and a clinical fellow (PN) with extensive experience in the management of SLE based on ‘real-life’ clinical scenarios. The initial draft was piloted on additional rheumatologists experienced in the management of SLE (JR, SS) and then adjusted. After alterations the final survey was reviewed, agreed by all and disseminated as an internet-based survey (Select Survey.NET) aimed at clinicians involved in the management of SLE. Thirty case scenarios were constructed to reflect a range of potential ‘states’ of clinical remission. These were grouped into seven ‘stems’ with one stem representing minor organ involvement—for example, joint and skin only; the other six stems represented patients with major organ involvement—for example, renal and neuropsychiatric (see online supplementary data 1). In all cases the patient was said to have “no evidence of any organ involvement, have a normal eye examination and a normal full blood count, renal profile, inflammatory markers and urinary examination”. Within each stem we then added variations based on duration of remission (1, 3 or 5 years), current serological abnormalities (presence or absence of high anti-dsDNA antibodies and/or low/normal complement levels), number of previous flares (single or multiple) and currently prescribed treatment (hydroxychloroquine (HCQ), steroids and/or ISS agents). Respondents were asked to indicate whether they would continue, reduce or withdraw each medication type in each scenario.

10.1136/lupus-2016-000173.supp1 supplementary data

#### Minor organ involvement stem

A 26-year-old woman with a 10 year history of SLE (rash, arthritis) with no clinical disease activity for the past 5 years was the model to assess how clinicians would deal with a patient who had only ever had mild disease and no evidence of clinical disease activity for a long period of time. A key aim of this ‘stem’ was to determine what low-dose treatment clinicians would use in such patients. We devised six associated scenarios comprising combinations of serological abnormality (complement low/normal and/or anti-dsDNA high/normal either alone or in combination) and one of three types of current medication regimens:

1. HCQ 200 mg alone;
2. HCQ 200 mg plus prednisolone 5 mg;
3. HCQ 200 mg, prednisolone 5 mg and methotrexate (MTX) 7.5 mg weekly.

#### Major organ involvement stems

A 26-year-old woman with a previous history of lupus nephritis (class IV) and neuropsychiatric involvement was the subject of the scenario for the major organ stems. A total of 24 scenarios were created reflecting combinations of the number of previous flares (single vs multiple), duration of remission (1, 3, 5 years) and serological abnormalities as outlined in the minor organ stem.

#### Participant recruitment

To identify clinicians with an interest in SLE, the survey link was sent to (i) corresponding authors of papers from *Lupus* journal published between January 2013 and December 2013 and (ii) international lupus working groups, including the British Isles Lupus Assessment Group, Systemic Lupus International Collaborating Clinics and the Thai Rheumatology Association.

Clinicians were asked about their specialty, duration of practice, number of patients with SLE seen each month and geographical location.

#### Statistical analysis

The number and proportion of respondents indicating whether to continue, reduce or withdraw each type of medication was calculated for each scenario. The association between case characteristics (number of flares, duration of absence of clinical disease activity, serological changes) and participant decisions about medication use was assessed using χ^2^ tests. Statistical tests were considered to be significant at p<0.05.

#### Ethics

This study was approved by the University of Manchester research ethics committee (reference number 13287). Anonymous online participation was taken as implied consent.

## Results

### Respondents

We contacted 185 clinicians, of whom 54 replied to our first email. After 2 weeks a second reminder email was sent to all participants. In total we received responses from 130 clinicians (response rate 70%), most of whom were rheumatologists (n=113/130, 86.9%). Response rates differed according to region, with a majority of responses from European (n=54, 41.5%) and Asian (n=53, 40.8%) physicians and a minority from North America (n=16, 12.3%). The median (range) duration of practice was 13 (2–42) years and 30 (2–200) patients with SLE were seen each month.

### Minor organ involvement scenario: approach to HCQ use

Physicians rarely withdraw HCQ whatever the clinical scenario. For example, in the scenario within stem 1 where the patient with previous mild disease was described as having a 5-year clinical remission, normal serology for the entire duration of that period and was taking HCQ 200 mg daily alone (ie, the mildest disease of all the scenarios), only 13% of physicians (n=16/120) indicated that they would withdraw the drug.

### Physicians preferred order for medication reduction in mild disease: approach to prednisolone

When HCQ was used in combination with steroids and/or MTX to control mild disease, it was also the least likely drug to be altered. In this scenario clinicians were more likely to maintain the current dose of HCQ and reduce either the steroid and/or MTX dose, with this trend being true for those with both normal and abnormal serology (see online supplementary data 1—Stem 1).

**Figure 1:**
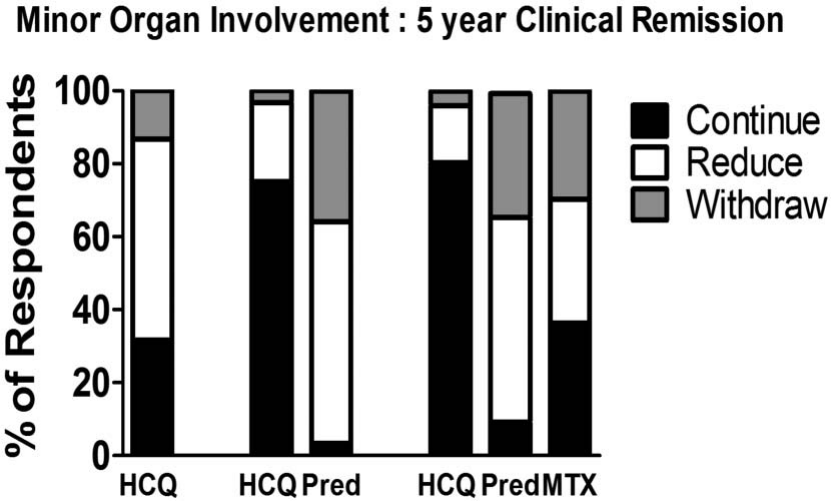
Pattern of alteration of medications by physicians in a patient with mild SLE (skin and joint only), no evidence of clinical disease activity for 5 years and current normal serology according to the patients’ baseline medication regimen. The three scenarios vary according to the drug combination the patient is taking. HCQ, hydroxychloroquine; MTX, methotrexate; Pred, prednisolone.

Prednisolone was the first medication that physicians suggested reducing or withdrawing during any prolonged remission, irrespective of whether it was used with HCQ alone or as part of a regimen involving prednisolone, HCQ and MTX. For example, after 5 years’ remission in a serologically quiescent patient, taking HCQ and prednisolone only, 116 (96.7%) preferred to reduce or withdraw steroids. Similarly, 110 (90.9%) preferred to alter steroids if used in combination with both HCQ and MTX for the same clinical scenario (figure 1).

### Major organ involvement: neuropsychiatric SLE, class IV lupus nephritis

#### Drug reduction or withdrawal

**Figure 2:**
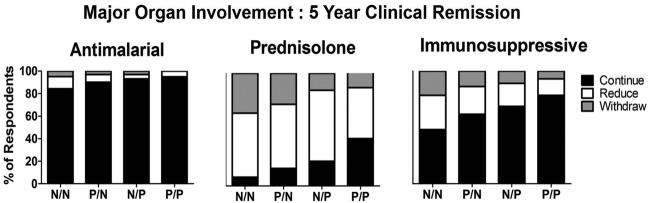
The pattern of alteration of HCQ, prednisolone and/or ISS drugs by physicians in a patient with a history of major organ involvement from their SLE (renal and neuropsychiatric) in clinical remission for 5 years with no evidence of current serological activity. The four scenarios vary according to the serological abnormality. N/N, normal dsDNA/normal complement; P/N, abnormal dsDNA/normal complement; N/P, normal dsDNA/abnormal complement; P/P, abnormal dsDNA/abnormal complement; HCQ, hydroxychloroquine; ISS, immunosuppressive.

For the management scenarios of patients with SLE with major organ involvement, the preferred order of reducing or withdrawing drugs was the same as for mild disease, with physicians most likely to suggest altering steroid doses first, followed by ISS agents, with AMs being reduced or withdrawn infrequently (figure 2). For example, (see online supplementary data 1—Stem 4) when a patient with previous major organ disease had been stable for 5 years and had normal serology, 94 (93.1%) respondents would plan to reduce or withdraw steroids, 53 (52.5%) would plan to reduce or withdraw the ISS and only 16 (15.8%) would reduce or withdraw HCQ.

#### Duration of stable disease

**Figure 3:**
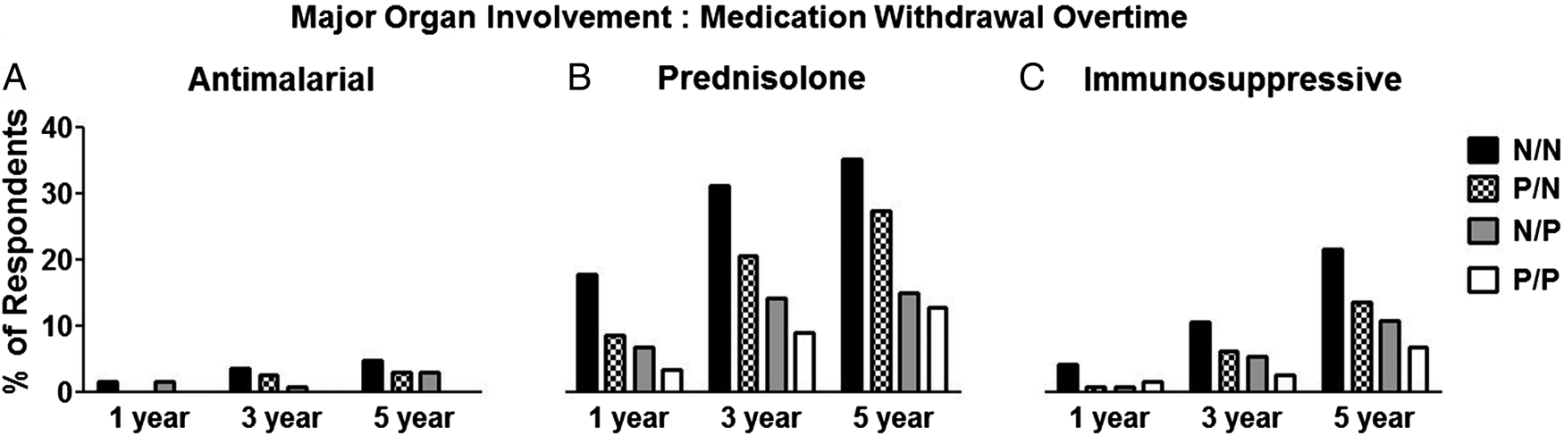
Longer duration of clinical remission is associated with increased likelihood of withdrawal of prednisolone and ISS drugs in a patient with SLE with previous major organ involvement and current normal serology. The four scenarios vary according to serological abnormality. N/N, normal dsDNA/normal complement; P/N, abnormal dsDNA/normal complement; N/P, normal dsDNA/abnormal complement; P/P, abnormal dsDNA/abnormal complement; ISS, immunosuppressive.

Where patients had stable, serologically inactive disease after a previous flare (stems 2–4), at 1 year the vast majority of physicians (92.4%, n=109/118) planned to reduce (74.6%, n=88) or completely withdraw (17.8%, n=21) steroids; this number was similar for remission duration of 3 or 5 years (3 years: 91%; 5 years: 93%). When specifically asked about *withdrawing* steroids from patients, physicians were more inclined to completely withdraw prednisolone treatment with longer duration of remission (figure 3).

In contrast, HCQ withdrawal or reduction was rarely initiated irrespective of the remission duration: a preference to reduce or withdraw HCQ was stated by only 6 (8.5%) physicians at 1 year, 14 (12.5%) at 3 years and 16 (15.5%) at 5 years' stability for the same clinical scenario. Similarly, the vast majority of physicians opted to continue ISS agents across the times assessed. However, in those who did aim to withdraw ISS agents a longer duration of remission appeared to influence their intended practice—for example, 5 (4%) would withdraw ISS after 1 year of stable disease compared with 12 (11%) after 3 years and 22 (22%) after 5 years (figure 3).

#### Serological abnormalities

Serological abnormalities significantly influenced the physicians’ approach to treatment even in mild disease. For example, as outlined in the first scenario presented above, 104/120 (86.7%) physicians preferred to continue HCQ even after 5 years’ remission in mild disease, with 38 (31.7%) opting to continue HCQ at the same dose and 66 (55%) reducing the dose. Interestingly, when the same patient was described as having abnormal serology, a significant change in their intention towards treatment was noted despite the patient being in clinical remission for 5 years. Respondents were significantly less likely to taper the HCQ dose, with 82/117 (70.1%) continuing the same dose, 31 (26.5%) reducing the dose and only 4 (3.4%) aiming to withdraw the drug (p<0.01 vs previous scenario).

**Figure 4:**
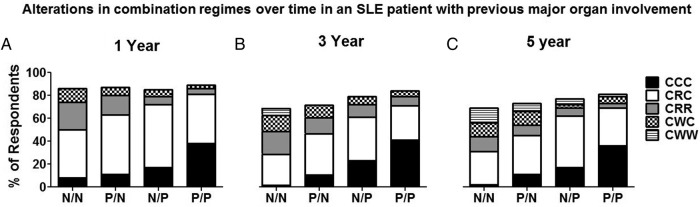
The pattern of alteration of HCQ, prednisolone and/or ISS drugs by physicians in a patient with a history of major organ involvement from their SLE (renal and neuropsychiatric) in clinical remission for 1, 3 or 5 years is influenced by the presence of current serological activity (stems 2–4). The four scenarios vary according to the serological abnormality presented to the physician. N/N, normal dsDNA/normal complement; P/N, abnormal dsDNA/normal complement; N/P, normal dsDNA/abnormal complement; P/P, abnormal dsDNA/abnormal complement; ISS, immunosuppressive. Antimalarial agents, prednisolone, immunosuppressive drugs: CCC, continue, continue, continue; CRC, continue, reduce, continue; CRR, continue, reduce, reduce; CWC, continue, withdraw, continue; CWW, continue, withdraw, withdraw. HCQ, hydroxychloroquine.

As with mild disease, the presence of active serology had a significant influence on physicians' decisions across a range of scenarios in patients with a previous history of major organ involvement (figure 4). In particular, we found that having *both* low complement and high antibodies to dsDNA significantly influenced the future plans for all drug classes at all time-points, with the exception of HCQ at 1 year in patients who had a single major flare, where over 90% of physicians would plan to continue the same dose anyway (stem 2). For example, after 5 years of stable disease in a patient with a single major flare previously (stem 4), in the presence of *both* low complement and high antibodies to dsDNA, 5/102 (4.9%) physicians would suggest a reduction or withdrawal of HCQ compared with 16 (15.7%) when the serology was normal (p=0.048).

For steroids and ISS agents in the same scenario (stem 4) the rates of reduction or withdrawal when both serological abnormalities were present compared with normal serology were 58% (n=59) vs 92% (n=94) (p<0.001) and 22% (n=22) vs 52% (n=53), (p<0.001), respectively. Thus, more than 40% of clinicians preferred to continue steroids with almost 80% expressing a preference towards continuing additional ISS without dose reduction in serologically active (both anti dsDNA and low complement) clinically quiescent patients even when the duration of clinical remission was 5 years (figure 4).

Examination of the contribution of the individual serological abnormalities showed that low complement levels significantly affected a physician's decision about drug treatment in 14 of 18 scenarios. In contrast, 5 of a potential 18 drug changes were significantly influenced by having high antibodies to dsDNA as the only serological abnormality.

### Frequency of disease flare

We found that one previous major flare or multiple flares of SLE had no clear influence on drug treatment changes.

### Geographical origin of the physician

Prescribing for patients in remission, particularly of steroids, varied geographically. For example, in the case of stable, mild disease for 5 years, steroids would be stopped by 24 (48%) European respondents, 4 (28.6%) North American respondents and 10 (19.6%) Asian physicians (p=0.04). In addition, for severe disease, Europeans were more likely to continue HCQ than Asian physicians, who preferred to reduce dosage in most scenarios (table 1) as reflected by the scenario of the patient with previously severe disease, now stable for 5 years. In this case 40 (93.0%) European respondents would continue HCQ compared with 31 (73.8%) Asian physicians (p=0.02). In contrast, and similar to the results observed in mild disease, across every timepoint assessed European physicians would be more likely to stop steroid treatment than their Asian counterparts (1 year: 8/48 vs 4/49 (p=0.38), 3 years: 20/46 vs 5/47 (p<0.001), 5 years: 21/43 vs 7/42 (p=0.002)), highlighting a geographical variation in approach to medication withdrawal. Neither duration of practice nor the number of patients seen monthly by physicians was found to significantly influence clinical practice and the decision to discontinue or continue steroids or continue HCQ across the various stems analysed.

**Table 1:** Comparison of respondents practising in Europe and Asia who would continue, reduce or withdraw treatments in a patient with SLE with major organ involvement in remission and normal serology

	Europe	Asia	p Value
*Stable for 1 year*			
HCQ			
Continue	45/48 (93.8)	42/49 (85.7)	0.04
Reduce	1/48 (2.1)	7/49 (14.3)	
Withdraw	2/48 (4.2)	0	
Prednisolone			
Continue	3/48 (6.3)	5/49 (10.2)	0.38
Reduce	37/48 (77.1)	40/49 (81.6)	
Withdraw	8/48 (16.7)	4/49 (8.2)	
Immunosuppressant agents			
Continue	35/48 (72.9)	16/49 (32.7)	<0.001
Reduce	11/48 (22.9)	32/49 (65.3)	
Withdraw	2/48 (4.2)	1/49 (2.0)	
*Stable for 3 years*			
HCQ			
Continue	44/46 (95.7)	35/47 (74.5)	0.004
Reduce	0	10/47 (21.3)	
Withdraw	2/46 (4.3)	2/47 (4.3)	
Prednisolone			
Continue	8/46 (17.4)	3/47 (6.4)	<0.001
Reduce	18/46 (39.1)	39/47 (83.0)	
Withdraw	20/46 (43.5)	5/47 (10.6)	
Immunosuppressant agents			
Continue	23/46 (50)	17/47 (36.2)	0.07
Reduce	16/46 (34.8)	27/47 (57.4)	
Withdraw	7/46 (15.2)	3/47 (6.4)	
*Stable for 5 years*			
HCQ			
Continue	40/43 (93.0)	31/42 (73.8)	0.02
Reduce	1/43 (2.3)	9/42 (21.4)	
Withdraw	2/43 (4.7)	2/42 (4.8)	
Prednisolone			
Continue	5/43 (11.6)	3/42 (7.1)	0.002
Reduce	17/43 (39.5)	32/42 (76.2)	
Withdraw	21/43 (48.8)	7/42 (16.7)	
Immunosuppressant agents			
Continue	24/43 (55.8)	17/42 (40.5)	<0.001
Reduce	5/43 (11.6)	23/42 (54.8)	
Withdraw	14/43 (32.6)	2/43 (4.8)	

Results are shown as number (%).

Patient was said to be in remission based on “no evidence of any organ involvement, has a normal eye examination and a normal full blood count, renal profile, inflammatory markers and urinary examination”. Duration of remission was varied as indicated above (1, 3 or 5 years) with the patient having no current serological abnormalities (ie absence of high anti-dsDNA antibodies and normal complement levels) and receiving currently prescribed treatment (HCQ, steroids and immunosuppressant agents).

HCQ, hydroxychloroquine.

## Discussion

We studied clinicians' opinions on management of clinical remission in patients with SLE across a range of hypothetical scenarios with different organ involvement, duration of clinical remission and serological results. Our findings suggest that clinicians' preferred practice for continuation of medications in clinical remission are influenced chiefly by the presence of serological abnormalities, especially when patients have *both* low complement and high antibodies to dsDNA, a response that is primarily driven by low complement. We also identified clear preferences for the sequence in which drugs were altered. Physicians prefer first to reduce and withdraw steroids from any therapeutic regimen, followed by ISS drugs. HCQ is rarely discontinued. European physicians appear more inclined to withdraw steroid treatment altogether than their Asian counterparts.

Remission is the goal of SLE treatment.^5^ However, there is no international consensus on the definition of remission according to the influence of serology, disease duration and medications. Owing to this lack of standardisation, previous studies of remission in SLE have been characterised by a wide variety of definitions with a consequential disparity in reported remission rates.^3^ ^10–13^ Steiman *et al*^12^ reported that only 2% of patients had prolonged clinical remission at 5 years without steroid and ISS drugs in the Toronto cohort. In contrast, the 1-year remission rate was as high as 53% in an Italian study when only clinical criteria were used and serology and medications were not taken into account. This highlights the significant confounding influence of medications and serological investigations on any consensus definition of remission in SLE.^3^ ^13^

In our survey we found that even after 5 years of clinical remission in patients with a history of major organ involvement, 40% of physicians preferred to continue steroids and 80% ISS unaltered if any evidence of active serology was seen. It might be argued that any beneficial effect from continued medication administration in preventing flares in this population might be outweighed by the disadvantages of prolonged use of fixed treatments, all with well-recognised side effects, without obvious clinical need. Steiman *et al* followed up patients who had sustained serologically active, clinically quiescent disease for more than 2 years and reported that approximately 40% continued to be clinically quiescent throughout the follow-up period.^14^ Our observation of physician behaviour therefore suggests that a population of patients with SLE exists for whom continued use of treatment may not have any real benefit. Further studies and trials are needed to determine the true risks and benefits of continuing or withdrawing treatment and current clinical trials, such as the National Institute of Allergy and Infectious Diseases sponsored mycophenolate mofetil withdrawal in SLE study (NCT 01946880), will help to inform future clinical practice in much the same way that the Canadian Hydroxychloroquine Study Group demonstrated the beneficial effect of AMs in the disease.^15^ ^16^

Our study showed that steroid reduction and withdrawal is the most likely choice of physicians, irrespective of the clinical scenario. The adverse effects of glucocorticoids are well established, particularly in patients with SLE, and our study suggests that physicians are aware of these risks and attempt to mitigate them as early as possible once disease control is achieved.^17^ Nevertheless, a significant proportion of patients appear to be kept on some dose of steroid by their treating physician despite clinical remission, particularly if there is a history of severe organ involvement. This has previously been observed by Walsh *et al*^18^ in their study of proliferative lupus nephritis, where approximately 30% of patients continued to receive some dose of steroid in all scenarios after successful induction treatment. Our study has highlighted key factors which determine the decision to continue or withdraw steroids. A longer duration of remission appeared to have a modest effect on physicians’ treatment intention but the single biggest influence on any decision to adjust medication was the presence/absence of serological abnormalities, even when there was no clinical evidence of disease activity. Therefore, additional biomarkers, better than dsDNA and complement (which have a poor predictive value of impending flare) are urgently needed to assist treating physicians in identifying quiescent disease activity accurately and promptly allowing safe reduction of steroids.

We also noted some regional variation in prescribing practices, with European physicians having a greater tendency to continue HCQ and withdraw steroid doses than their Asian counterparts. Although disease severity across regions might affect this variation, this treatment pattern was also seen in the stem that reflected 5-year remission in a patient with mild skin and joint disease. In view of the significant contribution of steroids to the excess morbidity and mortality in SLE, this observation warrants further investigation, especially as failure to reduce/withdraw steroids beyond prespecified endpoints can be deemed as ‘treatment failure’ in clinical trials, which are often conducted across large geographical regions.^19^ ^20^

Current guidelines recommend using long-term AMs in all patients with SLE unless contraindicated.^5^ Our data indicate that this is now standard practice across the geographical spread of our survey, albeit with Asian physicians more likely to taper them over time. The benefits of AMs include reduction of flares, improvement of skin manifestations, prevention of damage and possibly reduction in mortality.^16^ ^21^ Therefore, while future guidelines and recommendations for definition(s) of remission must be evidence based, any definition that includes ‘drug-free’ patients will be a rare event as the majority of doctors continue to prescribe AMs long-term, even for those with only mild disease previously. AMs are also increasingly recognised as having beneficial effects beyond disease control and damage prevention. They may protect against thrombosis and loss of bone mass, improve lipid profiles and improve maternal outcomes during pregnancy.^21^ Thus physicians may choose to continue long-term AMs for reasons beyond disease control. Although our survey did not seek specifically to examine these issues it does highlight that any future definition of remission needs to bear in mind what doctors do in the real world. Our study indicates that co-treatment, duration of clinical remission and, in particular, serological activity need to be considered in any proposed definition(s) of remission. These three factors influence treatment decisions in patients with no apparent clinical disease manifestations and the more restrictive the definition(s) are for these variables, the smaller the pool of patients to study. One solution might be for a series of graded definitions of remission as suggested by both Franklyn *et al* and van Vollenhoven *et al*, with increasing rigour of remission definition taking into account disease duration, serology and AM use.^22^ ^23^ This approach is supported by our current data.

Our study has a number of limitations. We had to develop our own survey proforma as no standardised questionnaire, which could have been modified, was available. Although we had 30 scenarios, the variability that we might have introduced was restricted as a larger survey would have had a much lower response rate. We acknowledge that further understanding is required of the influence on decision-making of the exact order and sequencing of medication withdrawal, dose variation, nuancing of recency of flares, extent of serological abnormality and other organ involvement patterns, etc. We also recognise that such scenarios do not fully represent real-world practice where physicians will know their patients well and will better understand the waxing and waning of their disease. Also, we were not able to provide a systematic description of patients' history and thus the cases were somewhat theoretical. However, we did base cases on real scenarios and tried to vary several items in a standard way to understand how changing key items influences decision-making. In addition, not all participants responded to every question stem. Finally, given the number of respondents we did not adjust the data for multiple comparisons. However, given the clear trends observed across all the stems, we think our results are generalisable and reflective of real-world clinical practice.

In conclusion, we found that clinicians' approach to withdrawing or reducing treatment in patients with SLE in clinical remission varies substantially. Serological abnormalities and previous disease severity, in particular, influence a clinician's decision to reduce treatments, with HCQ rarely being withdrawn even after a prolonged period of remission in patients with only mild disease. It is unusual for clinicians to withdraw all treatments, even after a very prolonged period of clinical remission, potentially exposing patients to unwanted side effects from these medications. Any definition(s) of remission need to consider evidence on the way in which maintenance treatments are managed. Future clinical trials should also focus on determining best practice for when and how to safely withdraw treatment particularly in patients with serologically active, clinically quiescent disease.
