# Supplementary material for: Clinicians approaches to management of background treatment in patients with SLE in clinical remission: results of an international observational survey
Source: Lupus Sci Med. 2017 Jun 29;4(1):e000173. doi: 10.1136/lupus-2016-000173 (PMC5724341; doi:10.1136/lupus-2016-000173)
Supplement: supplementary data [file lupus-2016-000173supp001.pdf]

Supplemental Data

Minor Organ

Stem 1.

A 26-year-old woman has been diagnosed with SLE for 10 years. Her main features were arthritis, malar rash, lymphadenopathy and positive ANA and Anti-dsDNA. She has had no clinical disease activity for 5 years. Currently, she has no evidence of any organ involvement, has a normal eye examination and a normal blood and urinary examination. She has no side effect of current medications.

Current medication: hydroxychloroquine 200 mg daily

Antimalarial	Normal Serology	Active Serology	p
Continue	38(31.67%)	82(70.09%)	<0.001
Reduce	66(55%)	31(26.5%)	
Withdraw	16(13.33%)	4(3.42%)	
Continue	38(31.67%)	82(70.09%)	<0.001
Reduce/Withdraw	82(68.33%)	35(29.91%)	

Current medication: hydroxychloroquine 200 mg daily, prednisolone 5 mg daily

**Antimalarial**	**Normal Serology**	**Active Serology**	p
Continue	90(75%)	107(89.92%)	0.005
Reduce	26(21.67%)	12(10.08%)	
Withdraw	4(3.33%)	0	
Continue	90(75%)	107(89.92%)	0.002
Reduce/Withdraw	30(25%)	12(10.08%)	

Prednisolone	Normal Serology	Active Serology	p
Continue	4(3.33%)	34(28.57%)	<0.001
Reduce	73(60.83%)	72(60.50%)	
Withdraw	43(35.83%)	13(10.92%)	
Continue	4(3.33%)	34(28.57%)	<0.001
Reduce/Withdraw	116(96.67%)	85(71.43%)	

Current medication: hydroxychloroquine 200 mg daily, prednisolone 5 mg daily, MTX 7.5 mg weekly

Antimalarial	Normal Serology	Active Serology	p
Continue	97(80.17%)	106(87.6%)	0.245
Reduce	19(15.7%)	13(10.74%)	
Withdraw	5(4.13%)	2(1.65%)	
Continue	97(80.17%)	106(87.6%)	0.116
Reduce/Withdraw	24(19.83%)	15(12.4%)	

Prednisolone	Normal Serology	Active Serology	p
Continue	11(9.09%)	32(26.45%)	<0.001
Reduce	68(56.2%)	70(57.85%)	
Withdraw	41(33.88%)	19(15.7%)	
Continue	11(9.09%)	32(26.45%)	<0.001
Reduce/Withdraw	110(90.91%)	89(73.55%)	

MTX	Normal Serology	Active Serology	p
Continue	44(36.36%)	65(53.72%)	0.032
Reduce	41(33.88%)	31(25.62%)	
Withdraw	36(29.75%)	24(19.83%)	
Continue	44(36.36%)	65(53.72%)	0.007
Reduce/Withdraw	77(63.64%)	56(46.28%)	

Major organ

Stem2

A 26-year-old woman has been diagnosed with SLE for 2 years. Her main features were lupus nephritis class IV, neuropsychiatric involvement and positive ANA and Anti-dsDNA. She has had no clinical disease activity for 1 year. She was prescribed prednisolone, mycophenolate mofetil (MMF) and hydroxychloroquine at diagnosis and is currently taking prednisolone 7.5 mg once daily, mycophenolate mofetil(MMF) 2,000 mg daily and hydroxychloroquine 200 mg once daily. The current doses have been stable for 1 year. She has no evidence of any organ involvement, has a normal eye examination and a normal blood and urinary examination. She has neither corticosteroid nor MMF side effects.

	Normal serology	Anti dsDNA	Low complement	Both	p	P compare Normal Vs Both active serology	P compare Normal Vs antiDNA	P compare Normal Vs Com
**Antimalarial**								
Continue	91.53%(108)	94.02%(110)	94.07%(111)	95.76%(113)	0.595	0.179	0.460	0.446
Reduce	6.78%(8)	5.98%(7)	4.24%(5)	4.24%(5)				
Withdraw	1.69%(2)	0	1.69%(2)	0				
**Prednisolone**								
Continue	7.63%(9)	15.38%(18)	24.58%(29)	44.07%(52)	<0.001	<0.001	0.114	<0.001
Reduce	74.58%(88)	76.07%(89)	68.64%(81)	52.54%(62)				
Withdraw	17.8%(21)	8.55%(10)	6.78%(8)	3.39%(4)				
**MMF**								
Continue	56.78%(67)	73.5%(86)	82.91%(97)	86.44%(102)	<0.001	<0.001	0.006	<0.001

Reduce	38.98%(46)	25.64%(30)	16.24%(19)	11.86%(14)				
Withdraw	4.24%(5)	0.85%(1)	0.85%(1)	1.69%(2)				

Stem3

A 26-year-old woman has been diagnosed with SLE for 4 years. Her main features were lupus nephritis class IV, neuropsychiatric involvement and positive ANA and Anti-dsDNA. She has had no clinical disease activity for 3 years. She was prescribed prednisolone, mycophenolate mofetil(MMF) and hydroxychloroquine at diagnosis and is currently taking prednisolone 5 mg once daily, mycophenolate mofetil(MMF) 1,000 mg daily and hydroxychloroquine 200 mg once daily. The current doses have been stable for 3 years. She has no evidence of any organ involvement, has a normal eye examination and a normal blood and urinary examination. She has neither corticosteroid nor MMF side effects.

	Normal serology	Anti dsDNA	Low complement	Both	p	P compare Normal Vs Both active serology	P compare Normal Vs antiDNA	P compare Normal Vs Com
**Antimalarial**								
Continue	87.5%(98)	89.19%(99)	94.64%(106)	96.43%(108)	0.04	0.014	0.694	0.058
Reduce	8.93%(10)	8.11%(9)	4.46%(5)	3.57%(4)				
Withdraw	3.57%(4)	2.7%(3)	0.89%(1)	0				
**Prednisolone**								
Continue	10.71%(12)	18.02%(20)	30.36%(34)	46.85%(52)	<0.001	<0.001	0.207	0.001
Reduce	58.04%(65)	61.26%(68)	55.36%(62)	44.14%(49)				
Withdraw	31.25%(35)	20.72%(23)	14.29%(16)	9.01%(10)				
**MMF**								
Continue	44.64%(50)	62.16%(69)	69.64%(78)	77.68%(87)	<0.001	<0.001	0.010	<0.001
Reduce	44.64%(50)	31.53%(35)	25%(28)	19.64%(22)				
Withdraw	10.71%(12)	6.31%(7)	5.36%(6)	2.68%(3)				

Stem4

A 26-year-old woman has been diagnosed with SLE for 6 years. Her main features were lupus nephritis class IV, neuropsychiatric involvement and positive ANA and Anti-dsDNA. She has had no clinical disease activity for 5 years. She was prescribed prednisolone, mycophenolate mofetil(MMF) and hydroxychloroquine at diagnosis. MMF was replaced by azathioprine 5 years ago due to side effect. She is currently taking prednisolone 5 mg once daily, azathioprine 50 mg daily and hydroxychloroquine 200 mg once daily. The current doses have been stable for 5 years. Currently, she has no evidence of any organ involvement, has a normal eye examination and a normal blood and urinary examination. She has neither corticosteroid nor azathioprine side effects.

	Normal serology	Anti dsDNA	Low complement	Both	p	P compare Normal Vs Both active serology	P compare Normal Vs antiDNA	P compare Normal Vs Com
**Antimalarial**								
Continue	84.31%(86)	90.2%(92)	93.14%(95)	95.05%(96)	0.300	0.048	0.378	0.115
Reduce	10.78%(11)	6.86%(7)	3.92%(4)	4.95%(5)				
Withdraw	4.9%(5)	2.94%(3)	2.94%(3)	0				
**Prednisolone**								
Continue	7.84%(8)	15.69%(16)	22%(22)	42.16%(43)	<0.001	<0.001	0.116	0.017
Reduce	56.86%(58)	56.86%(58)	63%(63)	45.1%(46)				
Withdraw	35.29%(36)	27.45%(28)	15%(15)	12.75%(13)				

**AZA**								
Continue	48.04%(49)	61.76%(63)	68.63%(70)	78.43%(80)	0.002	<0.001	0.045	0.004
Reduce	30.39%(31)	24.51%(25)	20.59%(21)	14.71%(15)				
Withdraw	21.57%(22)	13.73%(14)	10.78%(11)	6.86%(7)				

Stem 5

A 36-year-old woman has been diagnosed with SLE for 15 years with frequent major organ flares. Her main features are lupus nephritis class IV, neuropsychiatric involvement as well as malar rash, pleurisy and arthritis, positive ANA and Anti-dsDNA. Prednisolone and hydroxychloroquine have been continuous since diagnosis. She has no clinical disease activity in the last year. Currently, she has had no evidence of any organ involvement, has a normal eye examination and a normal blood and urinary examination. Her current treatment are prednisolone 10 mg once daily, mycophenolate mofetil(MMF) 2,000 mg daily and hydroxychloroquine 200 mg once daily which have been stable for 1 year. She has neither corticosteroid nor MMF side effects.

	Normal serology	Anti dsDNA	Low complement	Both	p	P compare Normal Vs Both active serology	P compare Normal Vs antiDNA	P compare Normal Vs Com
**Antimalarial**								
Continue	91.74%(100)	90.83%(99)	95.41%(104)	98.18%(108)	0.084	0.031	0.808	0.275
Reduce	5.5%(6)	7.34%(8)	3.67%(4)	1.82%(2)				
Withdraw	2.75%(3)	1.83%(2)	0.92%(1)	0				
**Prednisolone**								
Continue	6.42%(7)	11.93%(13)	24.77%(27)	37.27%(41)	<0.001	<0.001	0.321	<0.001
Reduce	85.32%(93)	84.4%(92)	70.64%(77)	60.91%(67)				
Withdraw	8.26%(9)	3.67%(4)	4.59%(5)	1.82%(2)				
**MMF**								
Continue	69.72%(76)	77.98%(85)	84.91%(90)	93.52%(101)	<0.001	<0.001	0.093	0.005
Reduce	27.52%(30)	21.1%(23)	15.09%(16)	5.56%(6)				
Withdraw	2.75%(3)	0.92%(1)	0	0.93%(1)				

Stem 6

A 36-year-old woman has been diagnosed with SLE for 15 years with frequent major organ flares. Her main features are lupus nephritis class IV, neuropsychiatric involvement as well as malar rash, pleurisy and arthritis, positive both ANA and Anti-dsDNA. Prednisolone and hydroxychloroquine have been continuously used since diagnosis. She has had no clinical disease activity in the last 3 years. Currently, she has no evidence of any organ involvement, has a normal eye examination and a normal blood and urinary examination. Her current treatments are prednisolone 5 mg once daily, mycophenolate mofetil(MMF) 1,000 mg daily and hydroxychloroquine 200 mg once daily which have been stable for 3 years. She has neither corticosteroid nor MMF side effects.

	Normal serology	Anti dsDNA	Low complement	Both	p	P compare Normal Vs Both active serology	P compare Normal Vs antiDNA	P compare Normal Vs Com
**Antimalarial**								
Continue	79.46%(89)	88.39%(99)	91.89%(102)	94.64%(106)	0.013	0.003	0.168	0.024
Reduce	16.96%(19)	8.93%(10)	7.21%(8)	5.36%(6)				
Withdraw	3.57%(4)	2.68%(3)	0.9%(1)	0				
**Prednisolone**								
Continue	14.29%(16)	20.54%(23)	27.03%(30)	41.07%(46)	<0.001	<0.001	0.270	0.016

Reduce	57.14%(64)	58.04%(65)	58.56%(65)	50%(56)				
Withdraw	28.57%(32)	21.43%(24)	14.41%(16)	8.93%(10)				
**MMF**								
Continue	43.24%(48)	60.36%(67)	67.86%(76)	78.57%(88)	<0.001	<0.001	0.023	0.001
Reduce	43.24%(48)	32.43%(36)	27.68%(31)	18.57%(21)				
Withdraw	13.51%(15)	7.21%(8)	4.46%(5)	2.68%(3)				

Stem 7

A 36-year-old woman has been diagnosed with SLE for 15 years with frequent major organ flares. Her main features are lupus nephritis class IV, neuropsychiatric involvement as well as malar rash, pleurisy and arthritis, positive both ANA and Anti-dsDNA. Prednisolone and hydroxychloroquine have been continuously used since diagnosis. She has had no clinical disease activity in the last 5 years. Currently, she has no evidence of any organ involvement, has a normal eye examination and a normal blood and urinary examination. MMF was replaced by azathioprine 5 years ago due to a side effect. Her current treatments are prednisolone 5 mg once daily, azathioprine 50 mg once daily and hydroxychloroquine 200 mg once daily which have been stable for 5 years. She has neither corticosteroid nor MMF side effects.

	Normal serology	Anti dsDNA	Low complement	Both	p	P compare Normal Vs Both active serology	P compare Normal Vs antiDNA	P compare Normal Vs Com
**Antimalarial**								
Continue	80.36%(90)	85.71%(96)	91.96%(103)	93.69%(104)	0.023	0.008	0.458	0.026
Reduce	15.18%(17)	9.82%(11)	7.14%(8)	5.41%(6)				
Withdraw	4.46%(5)	4.46%(5)	0.89%(1)	0.9%(1)				
**Prednisolone**								
Continue	11.61%(13)	17.86%(20)	27.68%(31)	40.18%(45)	<0.001	<0.001	0.312	0.007
Reduce	57.14%(64)	58.93%(66)	52.68%(59)	45.54%(51)				
Withdraw	31.25%(35)	23.21%(26)	19.64%(22)	14.29%(16)				
**MMF**								
Continue	37.5%(42)	54.55%(60)	62.16%(69)	77.27%(85)	<0.001	<0.001	0.012	0.012
Reduce	39.29%(44)	31.82%(35)	27.03%(30)	16.36%(18)				
Withdraw	23.21%(26)	13.64%(15)	10.81%(12)	6.36%(7)				
